# Heterogeneity and plasticity of tumor-associated macrophages in oral squamous cell carcinoma: implications for diagnosis and tumor microenvironment characterization

**DOI:** 10.3389/fimmu.2026.1864227

**Published:** 2026-07-09

**Authors:** Manuel Olmos, Tobias Möst, Christopher-Philipp Nobis, Nicolai Oetter, Linus Winter, Christoph Vogl, Marco Kesting, Jutta Ries

**Affiliations:** 1 Department of Oral and Cranio-Maxillofacial Surgery, Friedrich-Alexander-Universität Erlangen-Nürnberg, Erlangen, Germany; 2 Friedrich-Alexander-Universität Erlangen-Nürnberg (FAU), Erlangen, Germany; 3 Deutsches Zentrum Immuntherapie (DZI), University of Erlangen-Nuremberg and Universitätsklinikum Erlangen, Erlangen, Germany; 4 Comprehensive Cancer Center Erlangen-EMN (CCC ER-EMN), Erlangen, Germany; 5 Comprehensive Cancer Center Alliance WERA (CCC WERA), Erlangen, Germany; 6 Bavarian Cancer Research Center (BZKF), Erlangen, Germany

**Keywords:** CD115, CD11c, CD163, CD68, macrophage infiltration, macrophage polarisation, OSCC, plasticity

## Abstract

**Background:**

Oral squamous cell carcinoma (OSCC) is the predominant histological subtype of oral cavity cancers, with a 5-year survival rate of approximately 50%. The tumour microenvironment, particularly macrophage infiltration and polarization, plays a critical role in tumour progression and patient prognosis. Models describing macrophages as either M1 (pro-inflammatory) or M2 (anti-inflammatory) are increasingly recognized as oversimplified, given the functional heterogeneity and plasticity of tumour-associated macrophages (TAMs). This study aims to evaluate the expression of macrophage markers CD68, CD163, CD11c, and CD115 in OSCC compared to normal oral mucosa (NOM), to assess their diagnostic and prognostic value.

**Methods:**

A cross-sectional study of 179 tissue samples (111 OSCC, 68 controls) analysed macrophage markers (CD68, CD163, CD11c, CD115) via real-time qPCR. Statistical tests included Mann–Whitney U, ROC analysis for diagnostic utility, Spearman’s ρ for correlations, and assessments of associations with prognosis and recurrence. Cut-offs for gene overexpression were based on ROC results and evaluated clinically.

**Results:**

All four markers showed significantly higher expression in OSCC compared to NOM (p < 0.001 for CD68, CD163, CD11c; p = 0.001 for CD115). ROC analyses demonstrated diagnostic AUCs of 0.69 (CD68), 0.78 (CD163), 0.81 (CD11c), and 0.66 (CD115), indicating poor, fair and good discriminative capacity, respectively. Overexpression of the genes defined by COP was significantly associated with malignancy (p < 0.01). Elevated CD68 and CD163 levels correlated with higher tumour grading (G2/G3), while increased CD11c expression was linked to nodal metastasis (p = 0.04). Strong positive correlations existed between CD115 and the other markers (ρ > 0.61, p < 0.001), supporting a model of macrophage heterogeneity and plasticity. Concurrent upregulation of pro-inflammatory (CD11c) and M2-associated (CD163) markers suggests a complex, dynamic TAM landscape rather than a simple M1/M2 dichotomy.

**Conclusions:**

Altered RNA expression of the macrophage cell surface markers CD115, CD68, CD163 and CD11c in OSCC, and their respective association with tumour grading, N-status and perineural sheath infiltration, may serve as a basis for future single-cell sequencing or immunohistological – multiplex immunofluorescence – studies. Further investigation of these markers could lead to promising insights into the modulation of the tumour immune microenvironment.

## Introduction

Oral and oropharyngeal cancers combined represent the sixth most common malignancy worldwide (1). Considering the different entities of oral cavity cancer, oral squamous cell carcinoma (OSCC) accounts for 92-95% of oral malignancies with the 5-year overall survival rate of OSCC patients being approximately 50% (2, 3). Over the past 30 years, the prognosis of this malignancy has not significantly improved (4).

Numerous clinical and preclinical studies have demonstrated the importance of macrophages for the prognosis of patients with various types of cancer (5). In OSCC, the tumour microenvironment (TME) is characterised by increased macrophage infiltration (6). Macrophage polarisation at early stages has been shown to be a potential prognostic marker for tumour outcome, with heavy infiltration of M2-polarised macrophages correlating with poor tumour outcome at early stages (T1/T2, N0) of OSCC (7). Additionally, the malignancy of the primary tumour correlated with the lymph node macrophage polarization (8).

However, recent publications emphasise that this model is too simplistic and does not fully reflect the functional diversity of tumour-associated macrophages (TAMs) (9, 10). While the classic M1-to-M2 polarisation model, which is often discussed in connection with OSCC, represents a simplified view, alternative or more advanced conceptual hypotheses and explanatory approaches now exist. These show that the classic dichotomy is insufficient to explain the complex role of macrophages in the TME. This becomes particularly clear when attempting to understand the relationships between inflammation, immune response, and tumour progression.

Recent studies suggest that macrophages in the TME do not exhibit a clear M1 or M2 status, but rather a continuum of activation states (10). Instead of a purely binary polarization, there are multiple intermediate forms and subgroups that overlap functionally. This is particularly relevant because different micro-signals act simultaneously, inflammatory mediators and tumour secretions can activate parallel programs, and macrophages can dynamically switch between states in a reversible manner. This also applies to oral carcinomas and is discussed in several immunological reviews (6, 9, 10). A modern view describes TAM heterogeneity, functional subtypes, and contextual effects instead of a simple M1/M2 shift (10, 11). However, it is important to emphasize that this hypothesis does not suggest that M1/M2 models are incorrect, but rather that they represent only extreme points on a more complex spectrum.

Pro-tumour inflammation may be caused by “modified” or atypical M1 phenotypes. Some studies report that M1-like macrophages promote tumour growth under certain circumstances (6, 12). Exosome-dependent signals from OSCC cells induce M1-like TAMs with pro-tumoural IL-6 amplification (6). Such M1-like cells can promote epithelial-mesenchymal transition (EMT) and invasive behaviour—a pro-tumour function despite “pro-inflammatory” cytokines. This contradicts the simplistic idea that ‘more M1 means anti-tumour’.

In light of the functional heterogeneity and plasticity of TAMs in OSCC, markers reflecting distinct aspects of macrophage differentiation and activation are of particular interest. Therefore, CD115, CD68, CD163, and CD11c were selected for further analysis.

CD115 or colony stimulating factor-1 receptor (CSF1R) belongs to the platelet-derived growth factor family (13). CSF1R protein is expressed at low levels on haematopoietic stem cells (14) and mainly on monocytes and macrophages (15), osteoclasts, myeloid dendritic cells (16), microglia (17) and Paneth cells (18). It is overexpressed in many tumour entities and on TAMs (19). Impaired expression of CSF1R may promote cancer cell proliferation, invasion, and metastasis.

CD68 is highly expressed by monocytes and by macrophages. It functions both as a cell adhesion molecule and as a cell type marker. CD68 is often used as a pan-macrophage marker and thus serves as a comparative marker to other M1 or M2 markers (20–22). Although CD68 itself is not an immune checkpoint molecule, it is commonly used to assess the presence and distribution of TAMs within the TME. Therefore, CD68 was included in this study to evaluate macrophage infiltration and to provide a basis for comparison with functionally relevant macrophage-associated targets such as CSF1R.

CD163 functions as a marker for cells of the monocyte/macrophage lineage (23). Regarding macrophage polarization, CD163 is the best recognized marker for M2 macrophages (24–26). Specific depletion of the CD163+ macrophages results in a massive infiltration of activated T cells and tumour regression for melanoma (27). Based on these findings, differential expression in OSCC warrants further investigation.

CD11c is found in high levels on most human dendritic cells, monocytes, macrophages, neutrophils and some B-Cells (28). M1 polarized tissue macrophages are reported to express the CD11c antigen (29–31). Previously, high expression of CD11c was thought to indicate a favourable prognosis for certain types of cancer (32).

Aim of the following study is to analyse the correlation between CD115, CD68, CD163, CD11c expression rates and tumour progression in OSCC and therefore to further investigate their value as prognostic markers and potential therapeutic targets. A novel aspect of this study is that CD115 is analysed in depth alongside established macrophage markers, and correlation- and ratio-based approaches are employed to characterise the myeloid landscape in OSCC beyond the classical M1/M2 model.

## Materials and methods

This study was designed as a cross-sectional analysis to evaluate the association between marker expression and tumour progression in OSCC. The study was performed in accordance with the Declaration of Helsinki. A positive vote of the local ethics committee is present (ethics application number: 3962) and all patients consented to their participation in written form. All samples were collected in the period from September 2017 to November 2021 at the Department of Oral and Cranio-Maxillofacial Surgery of the Friedrich-Alexander University Erlangen-Nürnberg.

A total of 200 samples were collected, of which 179 were included in the final analysis. Among these, 111 samples comprised tumour tissue from the oral mucosa of patients with primary OSCC. Only treatment-naïve patients with no prior history of malignancy were included. Exclusion criteria included being under the age of 18, known systemic inflammatory or autoimmune diseases, and ongoing immunosuppressive therapy. The remaining 68 samples were obtained from the oral mucosa of healthy patients who served as the control group. All patients underwent minor maxillofacial surgery or trauma surgery and did not suffer from any malignant disease. Healthy tissue (NOM group) was defined as non-inflamed and not malignant. The following exclusion criteria were designed to ensure that the control group was healthy and had not received any treatment that could affect the expression of genes relevant to the immune system. Those with inflammation of the oral mucosa, dysplasia, precancerous changes, periodontitis, existing tumour diseases, systemic immune system diseases, immunological diseases, or ongoing immunosuppressive therapies were excluded. Participants under the age of 18 were also excluded.

RT-qPCR was used to verify the differential expression of the macrophage-specific immune modulators CD115, CD163, CD68 and CD11c, thereby identifying their significance as regulators of the immune response and their relevance for OSCC progression.

### Isolation of total RNA from tissue

RNA isolation from RNAlater samples was performed using the PreCellys method (Precellys^®^ homogenizer, Bertin Instruments Company, Bertin, France) which is suitable for grinding, lysing and homogenising tissue samples. Rapid and efficient RNA extraction was then carried out using the miRNeasy Mini Kit (Qiagen, Hilden, Germany). All steps were performed according to the manufacturer’s instructions. The total mRNA quality and quantity was assessed using the Nano-Drop 3.3 ND1000 spectrometer and the associated ND-1000 software (Thermo Fisher Scientific Company in Waltham, MA, USA, v3.8).

### Quantitative real-time PCR (RT-qPCR)

To analyse the expression of the test genes (CD115, CD163, CD68 and CD11c), mRNA was transcribed into single-stranded cDNA using the High-Capacity cDNA Reverse Transcription Kit (Applied Biosystems, part of Thermo Fisher Scientific Inc., Waltham, MA, USA).

The expression rate was then analysed using RT-qPCR with specific primers. Amplification was performed using the PowerSYBR^®^ Green PCR Master Mix of Applied Biosystems™ (Thermo Fisher Scientific Inc., Waltham, MA, USA). The gene-specific primer sequences used are shown in **Table 1**.

**Table 1:** The selected primers for RT-qPCR expression analyses of the investigated target gens.

Gene	Accession-nr.	Forward primer	Reverse primer	TM	Amp
CD115	NM_001349736.1	GAGCGACGTCTGGTCCTATG	AGGATGCCAGGGTAGGGATT	60	75
CD 11c	NM_000887.5, NM_001286375	AACTTGGACACAGAGGAGCTG	TGGTTGGCAGCTGTTATCTTTTG	60	125
CD163	NM_004244.6	CTT GGG GTT GTT CTG TTG GC	CCT CTT GAG GAA ACT GCA AGC	60	92
CD68	NM_001251.3	TGG GTG GGA TCA TCT CCA GT	TAG GCT GTC TGC ACC AGT TG	60	100

The table shows accession number, forward and reverse primer, annealing temperature (TM) and amplicon (Amp) for each gene. Number of cycles totals to 40.

The following analysis and data acquisition was done using QuantStudio 6 Pro from Applied Biosystems™ (Thermo Fisher Scientific Inc., Waltham, MA, USA). All primers annealed at 60 °C. Relative expression of the CD115, CD163, CD68 and CD11c genes was evaluated using the normalised cycle threshold (ΔCT) value and the ΔΔCT method.

### Statistical analysis

The IBM SPSS Statistics 22 programme (IBM Inc., New York, USA) was used to analyse the collected data statistically. The CT value was normalised using GAPDH as internal Control (CT_Target_ – CT_GAPDH_ = ΔCT). These values were applied for statistical analysis. Shapiro–Wilk test was used to check the RT-qPCR data for normal distribution. Relative gene expression (RQ) was determined using the ΔΔCT method (ΔΔCT = ΔCT_Control_ – ΔCT_Test group_) and calculated in Microsoft Excel using the formula RQ = 2^–ΔΔCT^. The p-value was identified using the Mann–Whitney U test to compare the groups. A p-value ≤ 0.05 was considered statistically significant. The data are visualised using box-and-whisker plots. These plots show the median, interquartile range, minimum and maximum range of gene expression. The area under the Receiver Operating Characteristic (ROC) curve shows how well the respective marker can differentiate between the two groups. This is created by plotting the true positive rate (sensitivity) against the false negative rate (1 - specificity). The highest Youden index (Y) is calculated from the ROC curve (Y represents the highest possible specificity combined with the highest possible sensitivity). This index can be used to derive the optimal cut-off point (COP) for distinguishing between the two groups. New subgroups of the two test groups are then created above and below the COP, with a value below the COP indicating overexpression of the gene. The chi-squared test can then be used to determine whether there is a link between altered gene expression and OSCC diagnosis and progression. Finally, the positive (PPV) and negative predictive values (NPV) were calculated.

The expression rates of the different Genes were correlated using the Spearman-Test. A p-value < 0.05 was considered to be significant. Spearman’s ρ was interpreted according to Cohen et al. (ρ = 0.1 weak, ρ = 0.3 moderate, ρ = 0.5 strong correlation) and was used to describe the strength of the correlation (33). The results are visualised by scatter plots.

To evaluate differences in gene expression ratios between two genes across groups (tumour vs. healthy), ΔCT values were used. For each sample, ΔΔCT was calculated as the difference between the ΔCT values of the two genes (ΔΔCT = ΔCT_Gene1_ − ΔCT_Gene2_), representing relative expression on a logarithmic scale. Group comparisons were performed using the Mann–Whitney U test based on ΔΔCT values. Lower ΔΔCT values indicate higher relative expression of Gene1 compared to Gene2, whereas higher values indicate lower expression. Data distributions were visualised using ΔΔCT values (boxplots). For linearized representation of group differences, mean ΔΔCT values per group were calculated and transformed to relative quantities (RQ) using RQ = 2^−ΔΔCT^. Higher RQ values indicate higher relative expression of Gene1 compared to Gene2. All statistical analyses were conducted on ΔΔCT values, while RQ values were used solely for reporting linearized differences between group means.

The analysis of disease-free survival (DFS) and overall survival (OS) in relation to the expression of the markers was performed using the Kaplan–Meier method. Differences between the survival curves were statistically evaluated using the log-rank test. Survival times were reported in months; statistical significance was set at a p-value ≤ 0.05.

## Results

The Shapiro-Wilk test showed that the ΔCT values of CD68, CD163, CD11c and CD115 were not normally distributed within the groups (p ≤ 0.05). Therefore, only non-parametric tests were used for the statistical analysis.

### Histomorphological parameters and demographics

180 tissue samples in total, including a maximum of 111 patients for the test group (OSCC) and 68 volunteers as a control group (NOM), were collected. Demographic data and histomorphological parameters for all patients are presented in **Table 2**. The values in the table are given for the CD115 marker only. This is due to slight differences in sample numbers resulting from missing data.

**Table 2:** Number of cases, demographic and clinical data of the studied patient collective.

		Patients (OSCC)		Healthy individuals (NOM)	
		N	% of cases	N	% of cases
Number of cases		111		68	
Gender (*p* = 0.609)	Female	35	33.7	26	40.0
	Male	71	68.3	39	60.0
	Unknown	5		3	
Mean age ± SD (*p* ≤ 0.001)		63.08 ± 12.17		54.01 ± 20.09	
Range of age		31–93 years		18–88 years	
			Valid cases %		
Tumour status	T1/T2	63	61.2		
	T3/T4	40	38.8		
	Unknown	8			
N-status	N0	61	58.6		
	N+	43	41.4		
	Unknown	7			
Grading	G1	10	9.8		
	G2	52	51.0		
	G3	40	39.2		
	Unknown	9			
UICC status	Early	39	40.2		
	Late	58	59.8		
	Unknown	14			
Recurrence	No	71	69.6		
	Yes	31	30.4		
	Unknown	9			
Localisation	BM	4	5.3		
	FOM	24	32.0		
	Tongue	14	18.7		
	Alveolar crest	26	34.7		
	FOM/tongue	4	5.3		
	Palate	2	2.7		
	Jaw angle	1	1.3		
	Unknown	36			

For the OSCC patients, staging parameters are given: Tumour size (T, grouped into small = T1/T2 and large T3/T4), Nodes (N)-status grouped into N0 (lymph nodes not affected) and N1(lymph nodes affected), grading, clinical UICC stage (early = Stadium I/II, late = stadium III/IV)), occurrence of recurrence and localisation are shown. BM, buccal mucosa; FOM, floor of the mouth.

### Comparison of CD68, CD163, CD11c and CD115 expression in tissue between OSCC and NOM group

Data on the expression of CD68, CD163, CD11c and CD115 derive from RT-qPCR and are given as ΔCT values.

**Figure 1:**
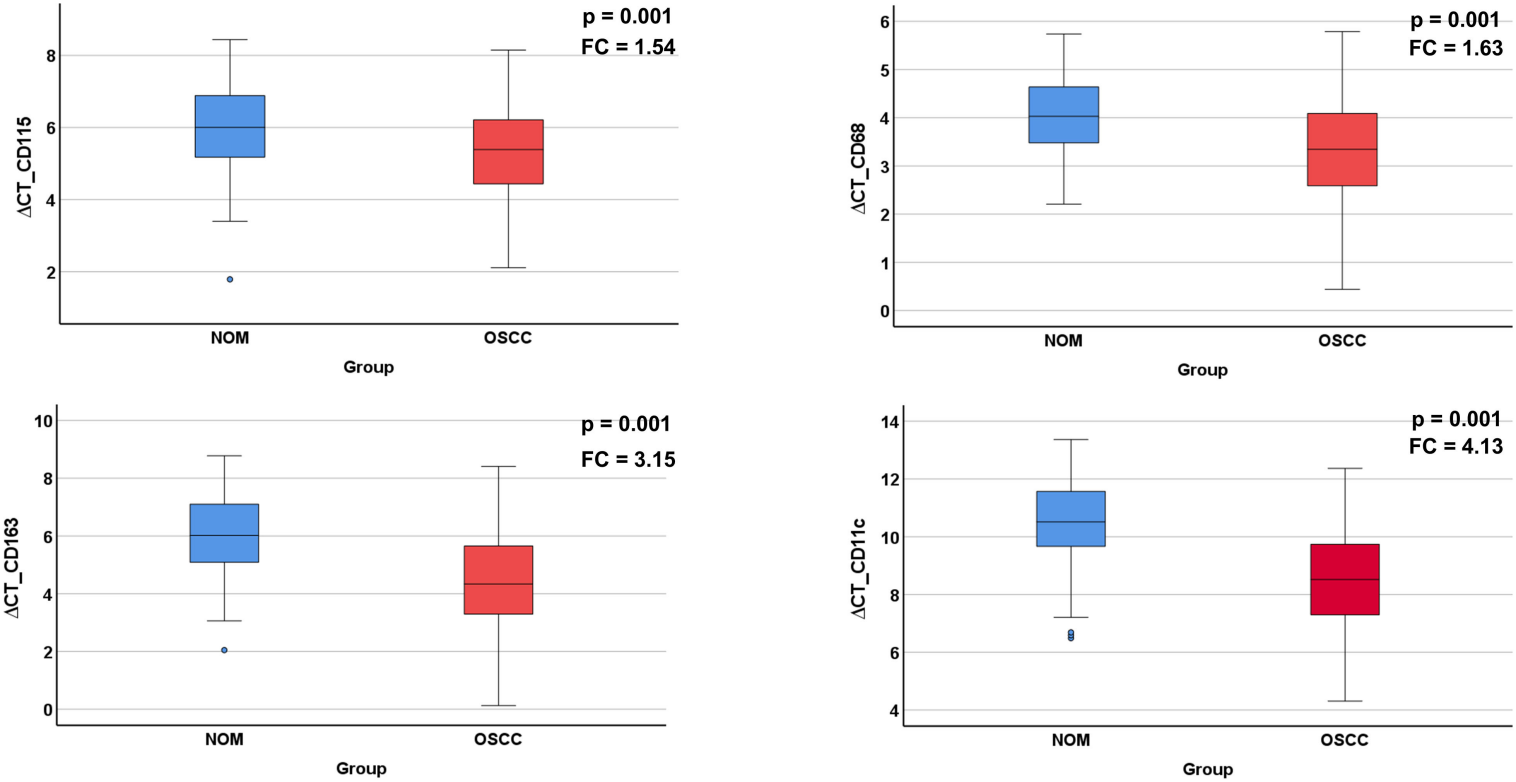
Increased expression of macrophage–associated transcripts in OSCC compared with NOM. Boxplots illustrating the distribution of RT-qPCR-derived ΔCT expression values for the macrophage markers CD115, CD68, CD11c and CD163, as well as their respective expression ratios, in OSCC and NOM. Expression levels are presented as ΔCT values, with lower values corresponding to higher transcript expression. Differences between OSCC and NOM were assessed using the Mann–Whitney U test.

Expression of CD68, CD163, CD11c and CD115 showed to be significantly increased in OSCC compared to NOM. The mean ΔCT values in OSCC amounted to 3.33 for CD68 (n = 110), 3.35 for CD163 (n = 111), 8.43 for CD11c (n = 109) and 5.32 for CD115 (n = 111). For NOM mean ΔCT values were detected at 4.03 for CD68 (n = 69), 6.00 for CD163 (n = 69), 10.47 for CD11c (n = 66) and 5.96 for CD115 (n = 68) (**Table 3**). Fold changes (FC) calculated by the ΔΔCT method revealed a prominent over expression in the OSCC group (FC_CD68_ = 1.63, FC_CD163_ = 3.15, FC_CD11c_ = 4.13, FC_CD115_ = 1.54). The Mann-Whitney U test showed statistical significance of differential expression between the groups OSCC and NOM (p_CD68_ < 0.001, p_CD163_ < 0.001, p_CD11c_ < 0.001 and p_CD115_ = 0.001). The results are summarised in **Table 3** and visualised in **Figure 1**.

**Table 3:** Comparison of the differential expression rates of CD68, CD163, CD11c and CD115 between the OSCC and NOM group.

		N	Mean ΔCT value	SD	FC	*p*-value MWU	*p*-value ROC	AUC value	Y	COP	SEN %	SPE %
CD68	**OSCC**	110	3.33	1.15	**1.63**	**< 0.001**	0.008	0.69	0.34	3.24	84.6	49
	**NOM**	69	4.03	0.81								
CD163	**OSCC**	111	3.35	1.64	**3.15**	**< 0.001**	0.001	0.78	0.44	4.65	84.6	59
	**NOM**	69	6.0	1.46								
CD11c	**OSCC**	109	8.43	1.81	**4.13**	**< 0.001**	0.001	0.81	0.51	9.82	72.3	80
	**NOM**	66	10.47	1.62								
CD115	**OSCC**	111	5.32	1.23	**1.54**	**0.001**	0.001	0.66	0.19	5.02	83.1	43
	**NOM**	68	5.96	1.23								

N, Number of cases; mean ΔCT value; SD, Standard deviation; FC, Fold change, Relative change in expression level between groups; p-value provided by MWU test, p-Value provided by ROC curves, AUC value, Area under the curve; Y, Youden´s index; COP, Cut off point; SEN, Sensitivity and SPE, Specificity for distinction of the two groups.

Bold characters represent the main focus of the corresponding tables.

**Figure 2:**
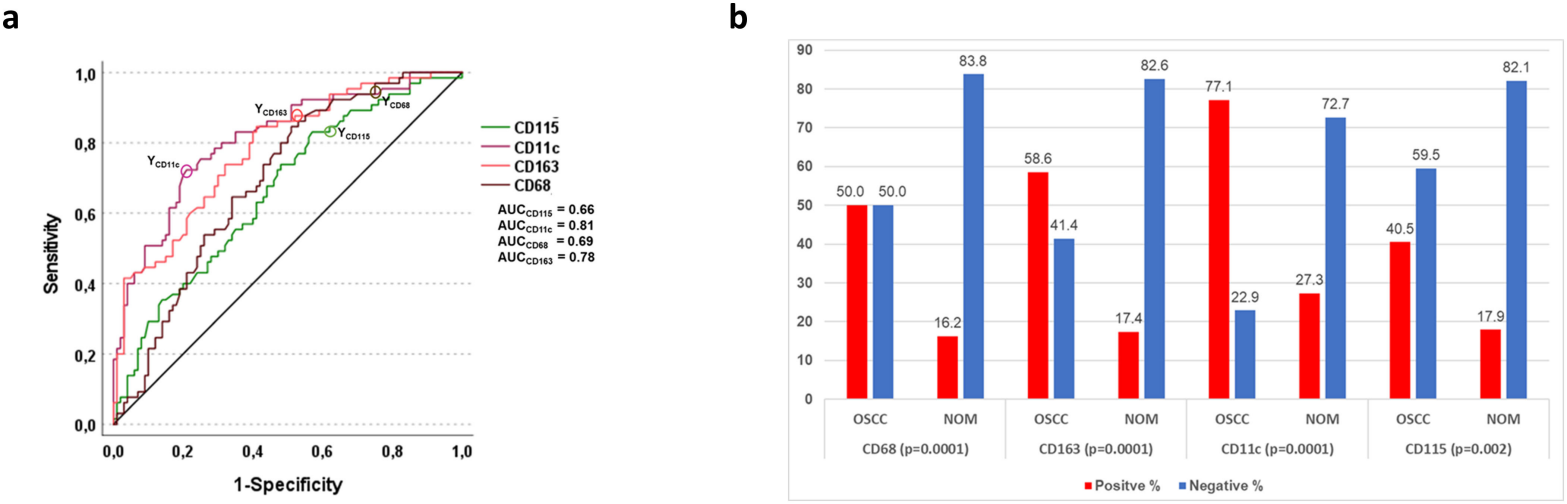
ROC analysis and COP-based chi-square classification of immune transcript expression distinguishing OSCC from NOM. **(a)** ROC analysis and COP-based chi-square classification of immune transcript expression distinguishing OSCC from NOM. **(b)** COP-based chi-square classification of immune transcript expression distinguishing OSCC from NOM.

To confirm statistical relevance, ROC curves were created and the area under the curve (AUC) was determined. AUC value for significance of upregulation of CD68, CD163, CD11c and CD115 was 0.69, 0.78, 0.81 and 0.66 respectively (**Table 3**, **Figure 2a**). Sensitivity (true positive rate) was found to be 84.6% for CD68, 84.6% for CD163, 72.3% for CD11c and 83.1% for CD115. The specificity (1- false positive rate) of CD68 over expression was 49%, that of CD163 was 59%, that of CD11c was 80%, and that of CD115 was 43% (**Table 3**). Thus, the ROC analysis demonstrated moderate to good discriminatory ability between OSCC and NOM tissue within the study cohort using the analysed markers. The highest AUC values were observed for CD11c (0.81) and CD163 (0.78), whilst the values for CD68 and CD115 were more moderate. Low specificity values for CD163 and CD11c limit clinical applicability.

Consequently, the optimal COP for distinguishing two groups, given as ΔCT values, was calculated for all four transcripts (**Table 3**, **Figure 2a**). The two groups (OSCC and NOM) were subdivided into positive and negative specimens using the generated COPs, to confirm that these parameters allow the detection of malignancy in a given sample. In this context, a ΔCT value lower than the COP threshold (indicating upregulated expression) was considered positive for malignancy.

Among the OSCC patients, 50% (57/114) exhibited increased expression of CD68, 58.6% (65/111) of CD163, 77.1 (84/109) of CD11c, and 40.5% (45/111) of CD115. By contrast, only 16.2% (11/68), 17.4% (12/69), 27.3% (18/66) and 17.9% (12/68) of the NOM samples exhibited increased CD68, CD163, CD11c and CD115 expression, respectively (**Table 4**). An expression level of CD68, CD163, CD11c and CD115 under the COP (positivity for overexpression) was significantly associated with malignancy (P = 0.001, 0.001, 0.001 and 0.002; **Table 4**, **Figure 2b**). The sensitivity for detecting malignancy was found to be 50%, with a specificity of 83.8% for CD68 positivity. CD163, CD11c and CD115 demonstrated sensitivities of 58.6%, 77.1% and 40.5%, and specificities of 82.6%, 72.7% and 82.1%, respectively (see **Table 4**, **Figure 2b**). Additionally, positive and negative predictive values for CD68, CD163, CD11c and CD115 are given in **Table 4**.

**Table 4:** Association between diagnosis and positivity of CD68, CD163, CD11c and CD115 expression in a specific tissue.

		N	Positive N/ %	Negative N/ %	p-value	*SEN* *%*	*SPE* *%*	PPV %	NPV %
CD68	**OSCC**	114	57 / 50	57 / 50	**0.001**	50	83.8	83.8	50
	**NOM**	68	11 / 16.2	57 / 83.8					
CD163	**OSCC**	111	65 / 58.6	46 / 41.4	**0.001**	58.6	82.6	84.4	55.3
	**NOM**	69	12 / 17.4	57 / 82.6					
CD11c	**OSCC**	109	84 / 77.1	25 / 22.9	**0.001**	77.1	72.7	82.4	65.8
	**NOM**	66	18 / 27.3	48 / 72.7					
CD115	**OSCC**	111	45 /40.5	66 / 59.5	**0.002**	40.5	82.1	78.9	45.5
	**NOM**	67	12 / 17.9	55 / 82.1					

A highly statistically relevant association between expression of CD68, CD163, CD11c and CD115 above the calculated threshold (COP) and malignancy was demonstrated. N, number of cases; p-value by χ^2^-test; SEN, Sensitivity and SPE; Specificity for diagnostic use between groups; PPV, Positive predictive value; NPV, Negative predictive value.

Bold characters represent the main focus of the corresponding tables.

### Association of differential expression of CD68, CD163, CD11c and CD115 in OSCC to prognostic parameters

**Figure 3:**
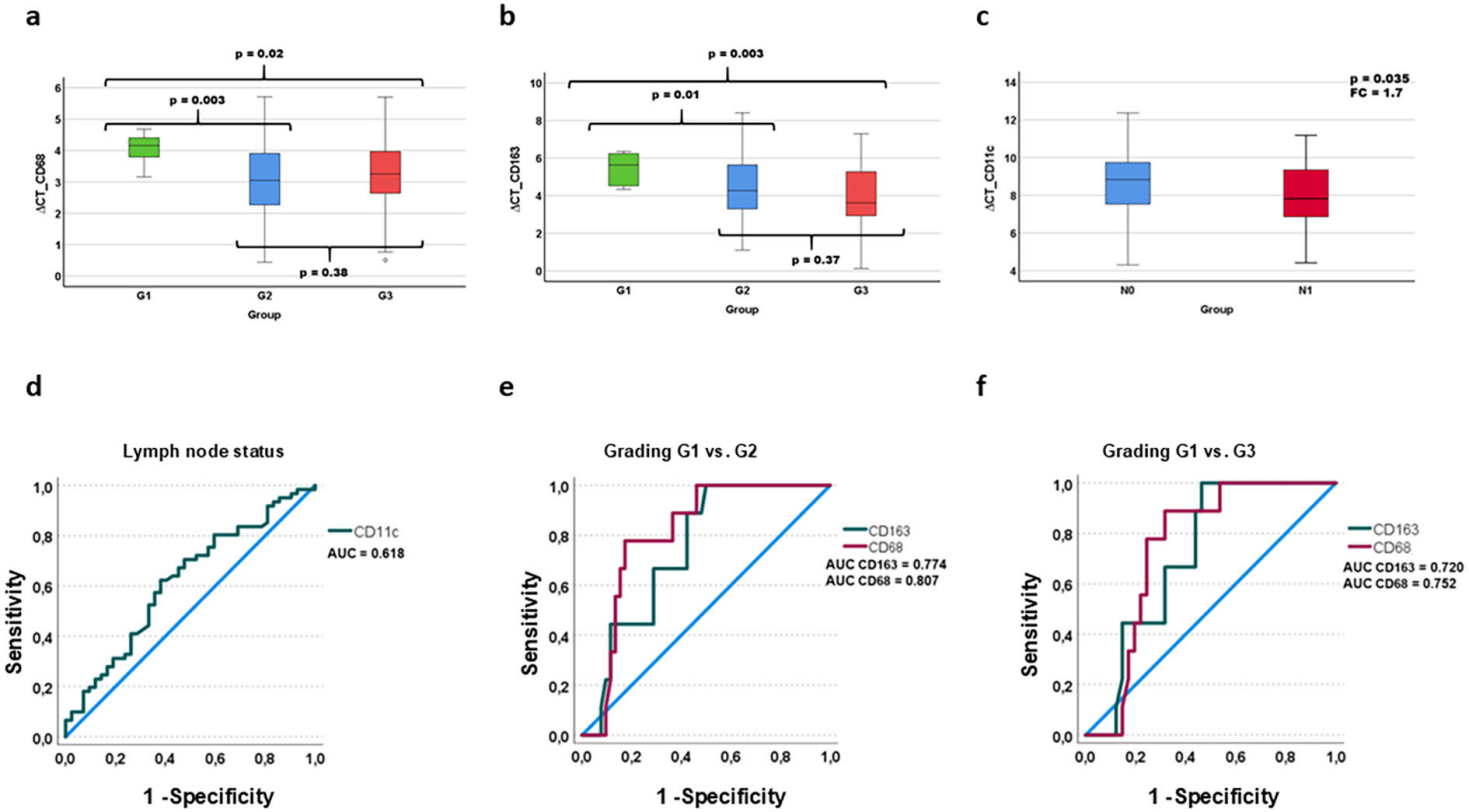
Association of differential macrophage transcript expression with clinicopathological parameters in OSCC. An association was observed between the differing expression levels of CD68 **(a)** and CD163 **(b)** and tumour grade, as well as between CD11c and positive lymph node status **(c)**. ROC analysis for CD11c to distinguish between lymph node status N0 and N1 showed low discriminatory ability **(d)**. **(e, f)** show ROC curves for CD163 and CD68 to differentiate G1 from G2 and G1 from G3, respectively. CD68 demonstrated higher diagnostic specificity than CD163 in distinguishing G1 from G2 and G1 from G3. Diagonal line = reference line.

Association of differential CD68, CD163, CD11c and CD115 expression in OSCC tissue compared to NOM regarding the histopathological parameters like tumour status (T1/T2), the N-status (grouped N0/N1), grading (1–3), UICC-status (grouped. early/late), occurrence of a recurrence (no/yes) and the localisation (buccal mucosa, floor of the mouth, tongue, alveolar crest, floor of the mouth and tongue, palate and jaw angle) was carried out by Mann-Whitney-U (MWU) and Kruskal-Wallis test. The increased expression of CD68 (p = 0.029) and CD163 (p = 0.025) was significantly associated with tumour grading. The subsequent analysis using the Mann-Whitney U test revealed significant differences in the expression levels of CD68 and CD163 between differentiated (G1) and undifferentiated (G2, G3) tumour tissues. Comparisons between G1 and G2 showed significant differences, with p-values of 0.003 for CD68 (**Figure 3a**) and 0.01 for CD163 (**Figure 3b**). Similarly, comparisons between G1 and G3 also demonstrated significant differences, with p-values of 0.02 for CD68 and 0.003 for CD163 (**Figures 3a, b**). No significant differences in expression were observed between moderately differentiated (G2) and undifferentiated (G3) tissues, with p-values of 0.38 for CD68 and 0.37 for CD163 (**Figures 3a, b**). Correction for multiple marker tests was applied. In addition, increased CD11c expression was significantly associated with affected N-status (PN0/N1 = 0.04; FC = 1.7, **Figure 3c**). None of the transcript variants (CD68, CD163, CD11c and CD115) were associated with any other prognostic parameter.

Mann-Whitney U tests revealed no significant differences in marker distribution between lymphatic vessel invasion (p CD115 = 0.822, p CD68 = 0.419, p CD163 = 0.860, p CD11c = 0.283) or venous invasion (p CD115 = 0.618, p CD68 = 0.430, p CD163 = 0.285, p CD11c = 0.827). Additionally, no significant differences in expression were detected between perineural invasion status and expression differences of CD115, CD163 and CD11c (p CD115 = 0.279; p CD163 = 0.075; p CD11c = 0.152). However, CD68 expression was significantly associated with the presence of perineural sheath infiltration (p = 0.020). Tumours with perineural sheath infiltration exhibited significantly higher CD68 expression than tumours without.

The ROC analysis to assess diagnostic accuracy showed that CD11c had a low discriminatory ability in distinguishing between lymph node statuses (AUC = 0.618, **Figure 3d**). The AUC values show that CD68 and CD163 could be used for differentiation between well differentiated and undifferentiated OSCCs (**Figures 3e, f**). The AUC values were 0.774 (CD163) and 0.807 (CD68) for G1 vs. G2, and 0.720 (CD163) and 0.752 (CD68) for G1 vs. G3. Hence, CD68 demonstrated higher diagnostic specificity than CD163 in distinguishing G1 from G2 (AUC = 0.807 (CD 68) vs. 0.774 (CD163)) and G1 from G3 (AUC = 0.752 vs. 0.720 CD163)).

### Spearman correlation analysis of CD68, CD163, CD11c and CD115

The expression data of CD68, CD163 and CD11c in all tissue samples (OSCC and NOM combined) were strongly correlated with that of CD115 (Spearman’s ρCD68 = 0.615, p < 0.001; Spearman’s ρCD163 = 0.805, p < 0.001; Spearman’s ρCD11c = 0.645, p < 0.001). Similarly, in OSCC, CD68, CD163 and CD11c expression data were strongly correlated with CD115 expression data (Spearman’s ρCD68 = 0.611, p < 0.001; Spearman’s ρCD163 = 0.806, p < 0.001; Spearman’s ρCD11c = 0.652, p < 0.001). Similarly, for NOM, CD68, CD163 and CD11c expression data were strongly correlated with CD115 expression data (Spearman’s ρCD68 = 0.535, p < 0.001; Spearman’s ρCD163 = 0.795, p < 0.001; Spearman’s ρCD11c = 0.533, p < 0.001).

Combined, CD11c expression in OSCC and NOM tissue showed a strong correlation with CD68, CD163 and CD115 expression (Spearman’s ρCD68 = 0.661, p < 0.001; Spearman’s ρCD163 = 0.729, p < 0.001; Spearman’s ρCD115 = 0.645, p < 0.001). In OSCC tissue, CD68, CD163 and CD115 expression were also strongly correlated with CD11c expression (Spearman’s ρCD68 = 0.684, p < 0.001; Spearman’s ρCD163 = 0.607, p < 0.001; Spearman’s ρCD115 = 0.652, p < 0.001). However, while there was a strong correlation between the expression of CD11c and CD163 in NOM (Spearman’s ρCD163 = 0.658, p < 0.001), the correlation between the expression of CD11c and both CD68 and CD115 was only moderate (Spearman’s ρCD68 = 0.373, p < 0.001; Spearman’s ρCD115 = 0.533, p < 0.001).

There was a strong correlation between CD68 and CD163 for OSCC (Spearman’s ρCD163 = 0.677, p < 0.001), as well as for OSCC and NOM combined (Spearman’s ρCD163 = 0.697, p < 0.001), and a moderately strong correlation in NOM (Spearman’s ρCD163 = 0.585, p < 0.001).

**Figure 4:**
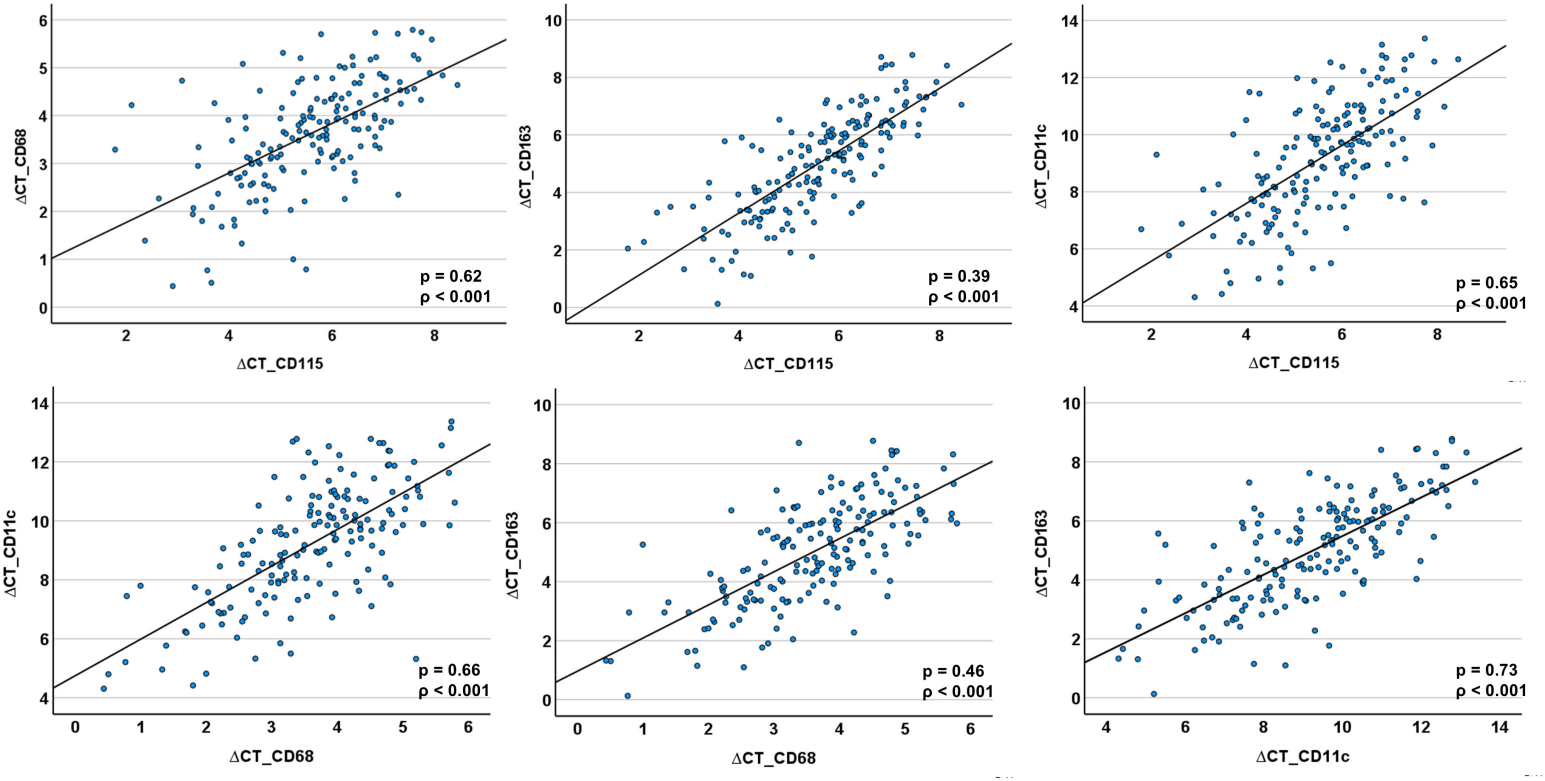
Spearman correlation analysis of the expression levels of the markers CD115, CD68, CD163, CD11c. Scatter plots illustrating pairwise correlations between transcript expressions of CD115, CD68, CD163 and CD11c in OSCC. The expression levels were derived from RT-qPCR measurements and are presented as ΔCT values. Spearman’s rank correlation coefficient (ρ) was utilised to assess the correlations. For each panel, the corresponding correlation coefficient and p-value are indicated within the plot.

The results have been summarised (**Table 5**) and presented for the entire patient cohort in **Figure 4**.

**Table 5:** Correlation between the expression rates of the macrophage markers CD115, CD68, CD163, CD11c.

		CD115	CD68	CD163
CD68	Spearman’s ρ	0.615^**^	–	0.234
	p-value	**< 0.001**	–	**0.008**
	N	169	–	129
CD163	Spearman’s ρ	0.388	0.463	–
	p-value	**< 0.001**	**< 0.001**	–
	N	131	133	–
CD11c	Spearman’s ρ	0.645^**^	0.661^**^	0.729^**^
	p-value	**< 0.001**	**< 0.001**	**< 0.001**
	N	173	167	174

For the correlation analysis, all samples were included (OSCC and NOM). Spearman’s correlations were calculated. Spearman’s ρ was used to describe the strength of correlation. Statistically relevant results are shown in bold. ** = The correlation is significant at the 0.01 level (two-tailed).

Bold characters represent the main focus of the corresponding tables.

### Association between changes in the expression ratios of two genes in OSCC compared with NOM

For the comparison between the two groups (tumour vs. healthy) using the MWU test ΔΔCT values were applied. For statistical analysis the ΔΔCT method was used. The relative differences in change between the groups were calculated using the formula RQ = 2 ^– ΔΔCTGene1–Gene2^.

**Figure 5:**
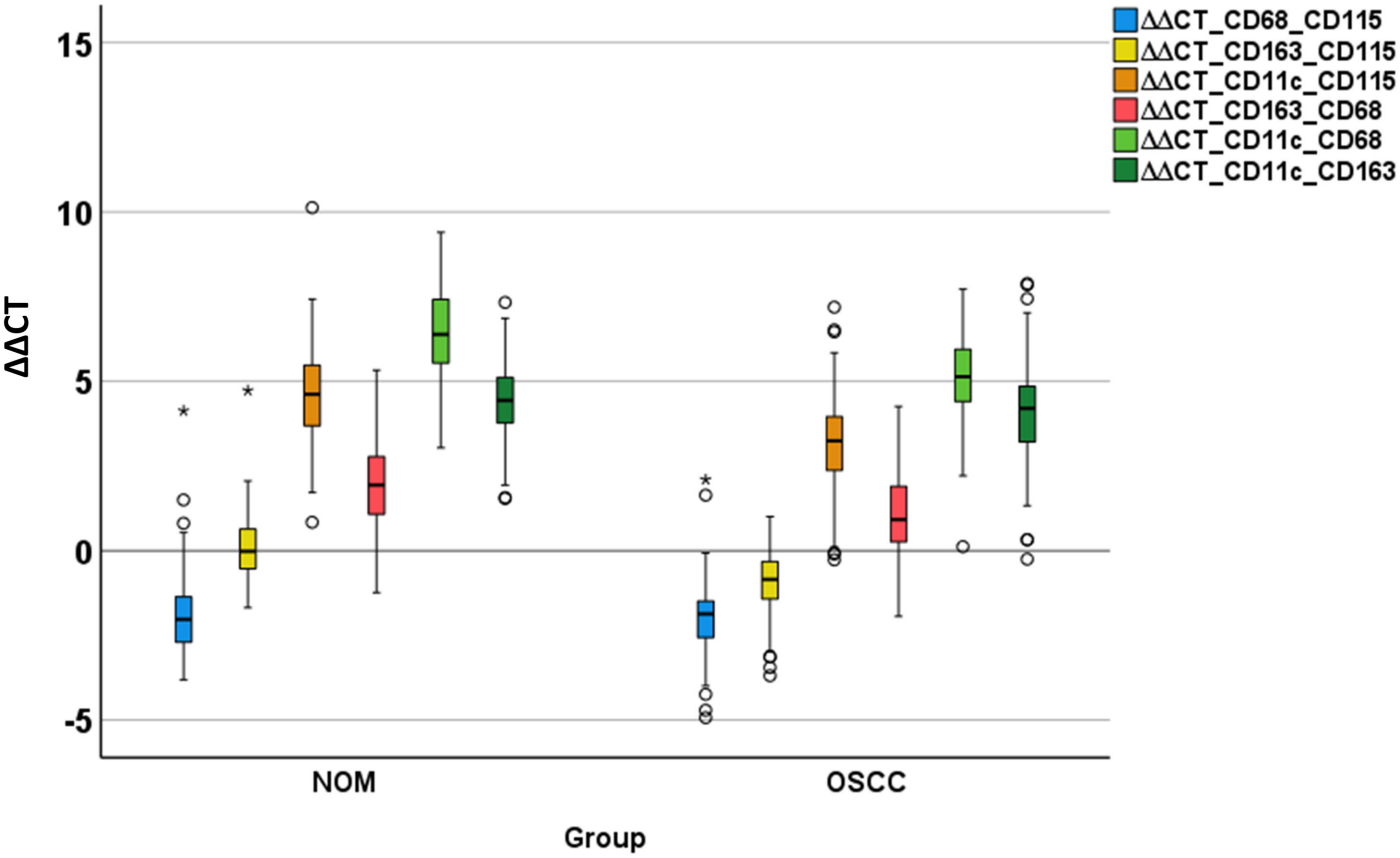
Relative expression of macrophage cell markers in OSCC versus healthy tissue. Boxplots show CD11c/CD115, CD163/CD115, CD11c/CD68, CD163/CD68, and CD68/CD115 in tumour and healthy oral mucosa. CD11c and CD163 to CD115 and CD68 ratios are significantly increased in tumours, while CD68/CD115 and CD11c/CD163 remain unchanged, indicating stable macrophage numbers with parallel activation of M1/dendritic-like and M2-like macrophages. Ratios are given as ΔΔCT values. Smaller ratios indicate a higher expression of gene1 compared to gene2.

In both OSCC and NOM, a low mean ΔΔCT (OSCC CD68_CD115: -1.97; NOM CD68_CD115: -1.80) and a high RQ (OSCC CD68_CD115: 3.92; NOM CD68_CD115: 3.49) value indicate higher CD68 expression. No significant difference was detected when the two groups were compared (p = 0.651). Comparing CD163 and CD115 expression, a low mean ΔΔCT (CD163_CD115: -1.00) and a high RQ (CD163_CD115: 2.00) value indicate higher CD163 expression in OSCC, whereas moderate mean ΔΔCT (CD163_CD115: 0.14) and RQ (CD163_CD115: 0.91) values indicate similar expression in NOM. A significant (p < 0.001) difference in expression rates between the two groups was detected. A high mean ΔΔCT (OSCC CD11c_CD115: 3.05; NOM CD11c_CD115: 4.52) and a low RQ (OSCC CD11c_CD115: 0.12; NOM CD11c_CD115: 0.04) value indicate lower CD11c expression compared to CD115 in both OSCC and NOM. A significant difference in expression rates was detected between the two groups (p < 0.001). In OSCC and NOM, a moderately high mean ΔΔCT (OSCC CD163_CD68: 1.04; NOM CD163_CD68: 1.94) and a moderately low RQ (OSCC CD163_CD68: 0.49; NOM CD163_CD68: 0.26) value indicate slight CD163 under expression compared to CD68. Significant difference was detected when the two groups were compared (p < 0.001). Comparing CD11c and CD68 expression, a high mean ΔΔCT (OSCC CD11c_CD68: 5.08; NOM CD11c_CD68: 6.34) and a low RQ (OSCC CD11c_CD68: 0.03; NOM CD11c_CD68: 0.01) value indicate lower CD11c expression in OSCC and NOM. Again, a significant (p < 0.001) difference in expression rates between the two groups was detected. Finally, comparing CD11c and CD163 expression, high mean ΔΔCT (OSCC CD11c_CD163: 4.07; NOM CD11c_CD163: 4.39) and low RQ (OSCC CD11c_CD163: 0.06; NOM CD11c_CD163: 0.05) values indicate lower CD11c expression in both OSCC and NOM. The difference in expression rates was significant (p < 0.001). The results are summarised in **Table 6** and visualised in **Figure 5**.

**Table 6:** The ratio of the expression levels of the various macrophage markers relative to CD115.

		N	Mean ΔΔCT value	SD	RQ	*P*-value MWU	AUC value
ΔΔCTCD68_CD115	**OSCC**	101	-1.97	1.09	3.92	0.651	0.51
	**NOM**	70	-1.8030	1.28	3.49		
ΔΔCTCD163_CD115	**OSCC**	109	-1.00	0.99	2.00	**< 0.001**	0.77
	**NOM**	70	0.1370	1.06	0.91		
ΔΔCTCD11c_CD115	**OSCC**	108	3.09	1.41	0.12	**< 0.001**	0.79
	**NOM**	67	4.5170	1.59	0.04		
ΔΔCTCD163_CD68	**OSCC**	103	1.04	1.19	0.49	**<0.001**	0.72
	**NOM**	70	1.9391	1.16	0.26		
ΔΔCTCD11c_CD68	**OSCC**	101	5.08	1.29	0.03	**< 0.001**	0.77
	**NOM**	67	6.3339	1.60	0.01		
ΔΔCTCD11c_CD163	**OSCC**	109	4.07	1.55	0.06	0.14	0.58
	**NOM**	66	4.3923	1.21	0.05		

ΔCT_Gene_, CT_Target_, CT_GAPDH_; ΔΔCT, ΔCT_Gene1_, ΔCT_Gene2_; RQ = 2 ^-ΔΔCT^, Smaller ΔΔCT values indicate higher expression of Gene1 relative to Gene2. RQ values were calculated using the mean ΔΔCT values of each group. A higher value indicates a higher expression because the values have been linearised.

Bold characters represent the main focus of the corresponding tables.

### Survival analysis

**Figure 6:**
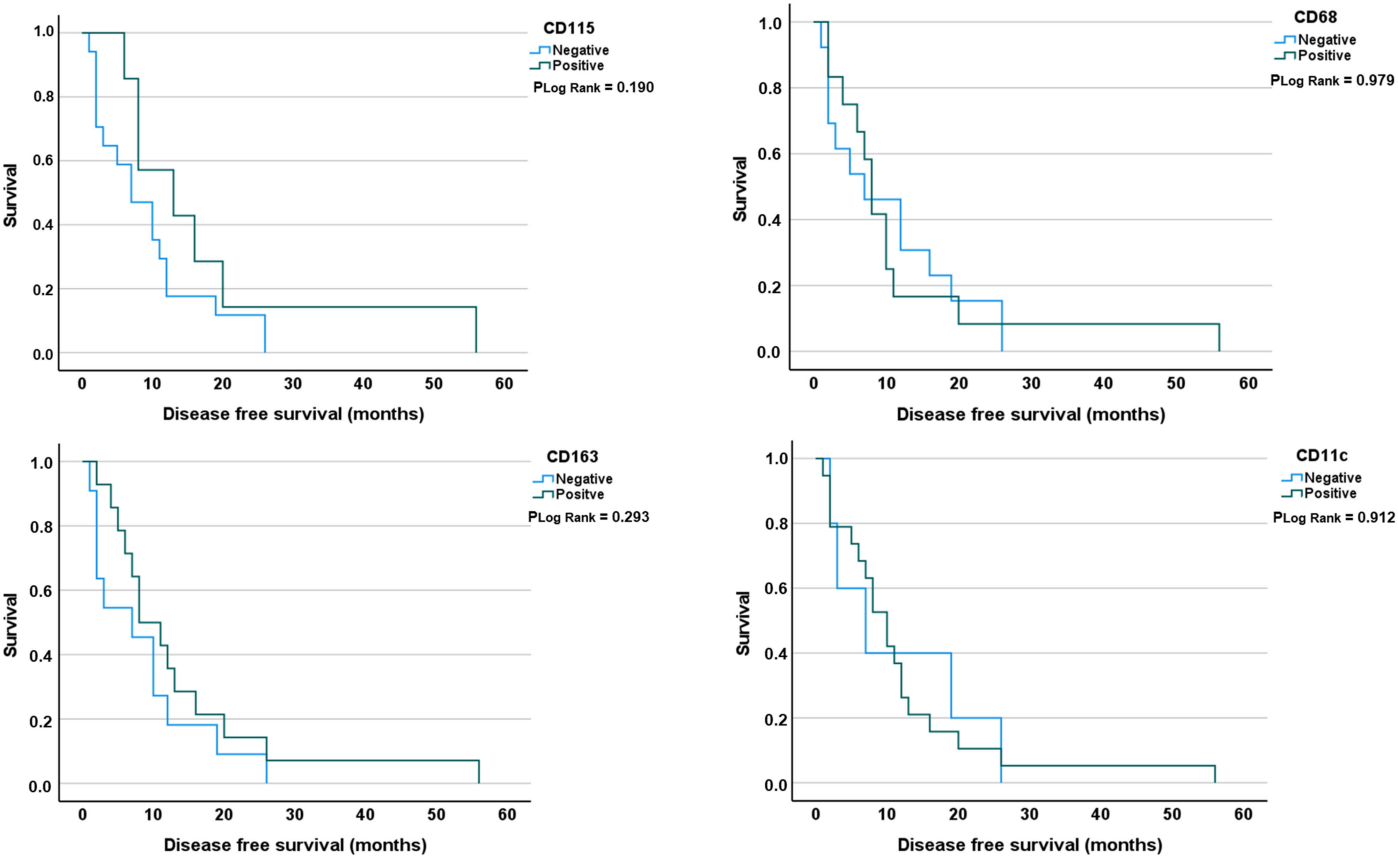
Kaplan–Meier analyses of disease-free survival (DFS) as a function of the expression of the markers CD115, CD68, CD163, and CD11c. The probability of survival is plotted on the y-axis, and disease-free survival time in months is plotted on the x-axis. The groups were compared using the log-rank test (p-values ≥ 0.05). Overexpression of none of the markers shows a statistically significant impact on recurrence-free survival.

**Figure 7:**
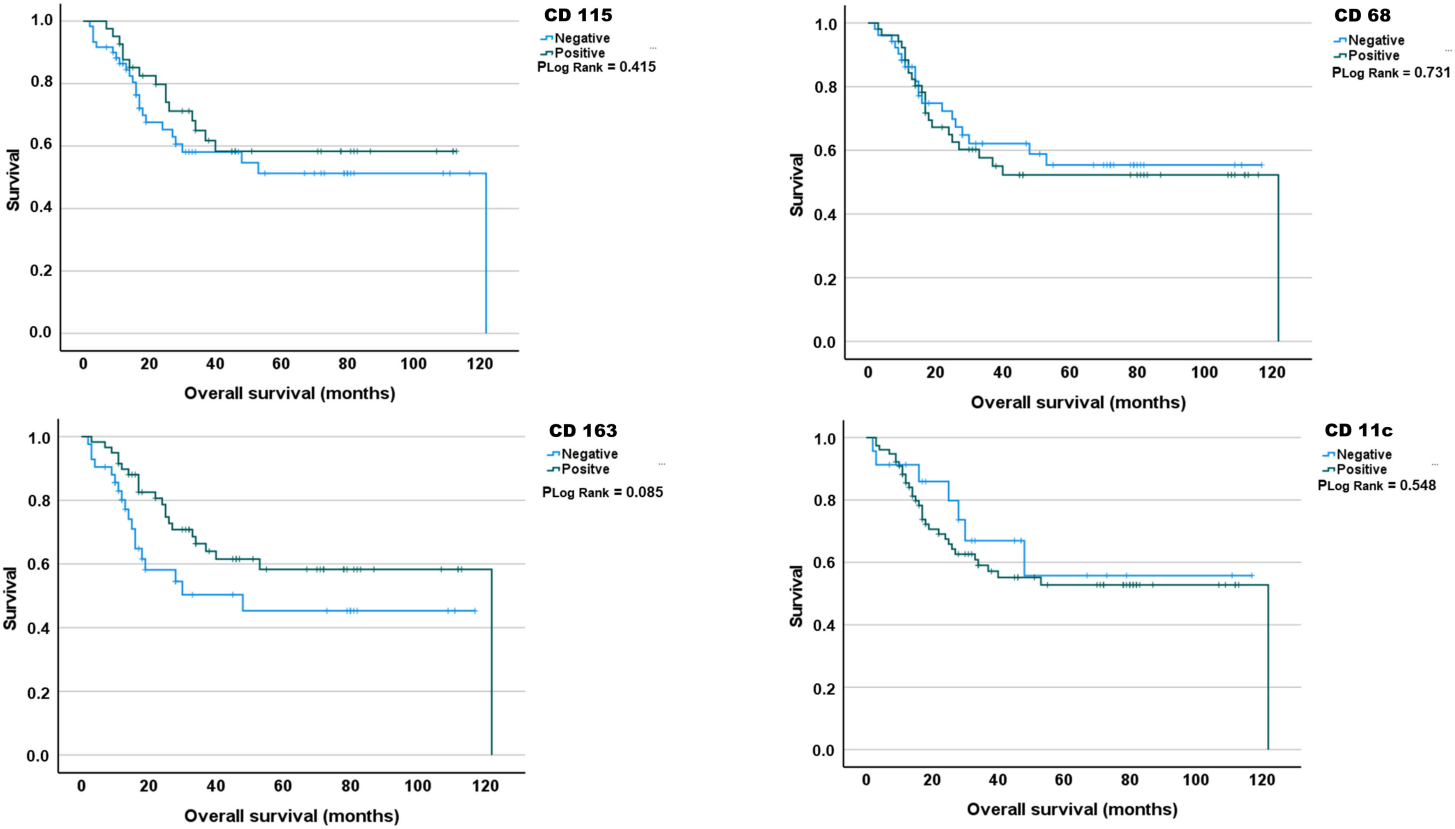
Kaplan–Meier analyses of overall survival (OS) as a function of the expression of the markers CD115, CD68, CD163, and CD11c. The probability of survival is plotted on the y-axis, and overall survival time in months is plotted on the x-axis. The groups were compared using the log-rank test (p-values ≥ 0.05). Overexpression of none of the markers shows a statistically significant impact on overall survival.

The Kaplan–Meier analyses show no statistically significant association between the expression of the markers and disease-free survival (DFS, **Figure 6**) or overall survival time (OS, **Figure 7**). All log-rank p-values were above the significance level of 0.05 (DFS: pCD115 = 0.190; pCD68 = 0.979; pCD163 = 0.293; p CD11c = 0.912; OS: pCD115 = 0.415; pCD68 = 0.731; pCD163 = 0.085; p CD11c = 0.548). Thus, no significant prognostic influence of the examined markers on disease-free survival or overall survival could be demonstrated.

## Discussion

In our study, we observed increased expression of CD68, CD115, CD163, and CD11c in OSCC tissues compared with non-inflamed healthy oral mucosa. Statistical evaluation showed significant association of overexpression with malignancy and indicates the ability for the detection of OSCC. However, the corresponding ROC analysis and AUC values do not allow conclusions to be drawn regarding clinical diagnostic utility and should therefore not be interpreted as evidence of this. Validation in independent, prospective cohorts, incorporating clinically relevant differential diagnoses such as precancerous lesions, is required for this purpose. Only then, conclusions regarding its clinical diagnostic value can be drawn.

Yet, although the altered expression levels of individual markers are not suitable for standalone diagnostic use due to their low specificity – with exception of CD11c-, their high sensitivity could make them valuable parameters in a multi-marker system. Furthermore, they could be integrated into an immune composite score to characterize the immune landscape of the tissue. This could support and improve the development and planning of immunotherapies aimed at modulating macrophage populations.

### Beyond the M1/M2 dichotomy

While previous reports describe a predominantly M2-polarized macrophage population (5, 7, 8), our data reveal co-elevated expression at the tissue level of both M2-associated (CD163) and pro-inflammatory myeloid subset (CD11c) markers. This discrepancy may be explained by the inherent plasticity of TAMs in OSCC. Chronic, subclinical inflammation in the oral cavity likely drives CSF1R/CD115-mediated monocyte recruitment, giving rise to a heterogeneous macrophage compartment that expresses both pro- and anti-inflammatory programs (6, 11, 34). This functional heterogeneity may mask clear correlations with most prognostic parameters, consistent with our findings, except for the association of CD11c expression with lymph node involvement, suggesting that pro-inflammatory or activated myeloid subpopulations may contribute to tumour invasion or metastatic dissemination. Overall, our results support a model in which OSCC TAMs are dynamically regulated and functionally flexible, reflecting the complex interplay between inflammation, tumour signalling, and immune modulation rather than a simple M1/M2 dichotomy. The most notable new aspect of this study is not the general concept of TAM plasticity itself, which has already been described in the literature (6, 9, 10, 12). Instead, it focuses on the combined analysis of the myeloid compartments associated with OSCC. To this end, expression analyses for CD115, CD68, CD163 and CD11c are being carried out in a large patient cohort. To our knowledge, CD115 has rarely been investigated in OSCC to date. Therefore, this study provides additional insights into CD115-dependent monocyte/macrophage recruitment as a potential target pathway within the TME. Furthermore, the combined correlation and ratio analyses revealed simultaneous activation of CD11c-associated pro-inflammatory and CD163-associated immunosuppressive networks, without any significant change in the total number of macrophages. This supports the concept of a functionally heterogeneous and dynamically regulated myeloid landscape in OSCC.

We hypothesize that chronic inflammatory signals in the OSCC TME drive sustained CSF1R (CD115)-dependent recruitment and expansion of myeloid cells, generating a heterogeneous macrophage compartment characterized by both pro-inflammatory (CD11c-associated) and anti-inflammatory (CD163-associated) features. This plastic myeloid landscape may simultaneously promote tumour progression and modulate local immune responses. Clinically, our findings suggest that therapeutic strategies targeting CSF1R signalling or reprogramming tumour-associated macrophages, rather than indiscriminate depletion, could more effectively modulate the tumour immune milieu and potentially enhance the efficacy of immunotherapy in OSCC patients.

In our analysis we used bulk RT-qPCR. However, this method does not provide single-cell resolution and therefore cannot fully capture the cellular heterogeneity and functional diversity of TAM populations in OSCC. Therefore, bulk PCR data in OSCC should always be supplemented by functional analyses, such as flow cytometry, immunohistochemistry, single-cell RNA-seq., spatial transcriptomics or co-culture assays. In particular, the growing evidence for hybrid TAM states suggests that transcriptional markers alone do not allow for reliable functional classification. Consequently, future studies utilizing single-cell RNA sequencing and spatially resolved multiplex immunofluorescence analyses should further improve our understanding of TAM heterogeneity in OSCC. Multiplex fluorescence staining enables the localization of specific immune cell populations within specific tumour compartments, the investigation of spatial interactions between different cell types, and the detection of co-expression of markers in individual cells. These analyses could significantly contribute to elucidating macrophage plasticity, functional cell states, and underlying signalling pathways within the TME in OSCC in the future.

### Association of differential expression of CD68, CD163, CD11c and CD115 in OSCC to prognostic parameters

Regarding the differential expression of the investigated genes and their association with prognostic parameters, the results obtained are consistent with the previously stated hypothesis of a more heterogeneous macrophage compartment. While, in line with current literature (20, 35), increased expression of CD68 and CD163 was significantly associated with tumour grading, CD11c overexpression was significantly associated with affected N-status indicating a more complex and non-dichotomic landscape for macrophage polarization. Bisheshar et al. recently identified perineural invasion as an important predictor for recurrence in OSCC (36). Our results further specify CD68 as a molecular marker as it was significantly overexpressed in tumours without perineural invasion.

### Changes in the expression ratios of two genes in OSCC compared with NOM

Our results indicate that the overall macrophage population in OSCC is quantitatively stable, as evidenced by the unchanged CD68/CD115 ratio compared to healthy tissue. However, the relative expression of CD11c and CD163 within this population is significantly increased, while the CD11c/CD163 ratio remains constant. This pattern suggests that OSCC induces a parallel functional activation of macrophages and dendritic cells, enhancing both proinflammatory (M1/dendritic-like) and immunosuppressive (M2-like) programs without altering the balance between subtypes. These findings highlight that the TME modulates macrophage function rather than abundance, supporting the concept that therapeutic strategies targeting macrophage activation states may be more effective than approaches aimed solely at reducing macrophage numbers.

### Spearman correlation analysis of CD68, CD163, CD11c and CD115

Spearman’s correlation analysis supports our hypothesis of a plastic myeloid landscape involving the CSF1R (CD115)-dependent recruitment and expansion of myeloid cells, as the expression data for CD68, CD163 and CD11c were strongly correlated with that for CD115 in OSCC, NOM and OSCC and NOM combined. The results indicate an environment featuring both anti- and pro-inflammatory characteristics and additionally stand in line with current trends in literature identifying CSF1R as a potential therapeutic target for cancer therapy (19, 37).

### Context of literature and limitations

Several published studies now demonstrate both similarities and significant discrepancies between transcriptional and functional macrophage phenotypes in OSCC and related HNSCC entities. This is particularly evident in the work of Xiao et al. and You et al., who demonstrated that OSCC-induced ‘M1-like’ TAMs, whilst expressing classic inflammatory markers such as TNFα, IL1β and IL6, possess pro-tumoral properties. Xiao et al. demonstrated that OSCC-derived exosomes induce an M1-like polarisation via THBS1, which paradoxically promotes the migration of OSCC cells (38). Building on this, You et al. demonstrated that the same TAMs, defined as ‘M1-like’ at the transcriptional level, promote epithelial-mesenchymal transition (EMT), stem cell-like properties, invasion and xenograft growth via the IL6/STAT3/THBS1 axis. These data illustrate that inflammatory gene expression profiles do not necessarily reflect anti-tumour functions (39).

In contrast, other studies report a stronger functional correlation between classical immunosuppressive TAM signatures and biological behaviour. In particular, CD163^+^, CD206^+^ or SPP1^+^ TAM subpopulations in HNSCC/OSCC consistently correlate with T-cell suppression, angiogenesis, invasion and a poor prognosis (40–43). This research supports the hypothesis that certain transcriptional programmes do indeed represent stable functional states, particularly in highly immunosuppressive TAM subpopulations.

Overall, however, the data available to date suggest that transcriptional markers alone are not sufficient to reliably define functional TAM states. Single-cell and spatial transcriptomic analyses increasingly point to continuous transitional states, hybrid activation programmes and strong context-dependent plasticity. Functional properties appear to be largely determined by spatial tumour niches, cell-cell communication and dynamic signals from the TME. Consequently, the direct functional validation of transcriptionally defined macrophage states – for example, through co-culture systems, proteomics, metabolic analyses and *in vivo* models – remains a key challenge. Future studies should therefore integrate single-cell transcriptomics with spatial omics, proteomics and functional perturbation assays to better define the biological relevance of TAM states in OSCC. In particular, systematic efforts are needed to determine which transcriptionally defined macrophage programs truly correspond to stable functional phenotypes and which merely reflect transient activation states within the dynamic TME. Additionally, future studies incorporating protein-based approaches such as multiplex immunofluorescence will be important for validating marker expression at the protein level, enabling the spatial localisation of positive cells, and further investigating co-expression patterns and the plasticity of macrophages within the TME. Such analyses could contribute significantly to a better understanding of the functional role of specific myeloid cell populations and the underlying signalling pathways at the single-cell level within the OSCC TME.

While our cohort is relatively large (n = 170), subgroup analyses for tumour stage, grading, or clinical outcome were beyond the scope of the current study and could provide additional mechanistic insights in future investigations. Despite these limitations, the study provides a comprehensive overview of myeloid infiltration and highlights the dynamic plasticity of TAMs in OSCC.

### Strengths

This study has several notable strengths. Most importantly, the relatively large cohort (n = 170) enhances the statistical robustness and generalizability of our findings within OSCC. In contrast to studies focusing on single polarization markers, we simultaneously assessed CD68, CD163, CD11c, and CD115 (CSF1R), enabling a broader characterization of macrophage infiltration, myeloid activation, and monocyte recruitment pathways. Another strength of this study is that it includes CD115 in its investigation of established macrophage markers, employing additional correlation- and ratio-based analyses. This provides a more comprehensive characterisation of the myeloid cell landscape in OSCC and offers an alternative to the classic M1/M2 model.

Collectively, the sample size and integrative marker approach strengthen the biological relevance of our findings.

## Conclusion

Altered RNA expression of the macrophage cell surface markers CD115, CD68, CD163 and CD11c in OSCC may serve as a basis for future single-cell sequencing or immunohistological – multiplex immunofluorescence - studies. Furthermore, the association of CD68 and CD163 overexpression with tumour grading and CD11c overexpression with N-status may provide useful insight into the immunological tumour microenvironment. Along with CD68 expression being significantly associated with the presence of perineural sheath infiltration, further investigation of these markers could lead to promising insights into the modulation of the tumour immune microenvironment.

## Data Availability

The raw data supporting the conclusions of this article will be made available by the authors, without undue reservation.
